# Artificial intelligence for precision medicine in neurodevelopmental disorders

**DOI:** 10.1038/s41746-019-0191-0

**Published:** 2019-11-21

**Authors:** Mohammed Uddin, Yujiang Wang, Marc Woodbury-Smith

**Affiliations:** 1 Mohammed Bin Rashid University of Medicine and Health Sciences, Dubai, UAE; 2 The Centre for Applied Genomics, The Hospital for Sick Children, Toronto, ON Canada; 3 Institute of Neuroscience, Newcastle University, Newcastle upon Tyne, UK; 4 School of Computing, Newcastle University, Newcastle upon Tyne, UK

**Keywords:** Neurological disorders, Biotechnology

## Abstract

The ambition of precision medicine is to design and optimize the pathway for diagnosis, therapeutic intervention, and prognosis by using large multidimensional biological datasets that capture individual variability in genes, function and environment. This offers clinicians the opportunity to more carefully tailor early interventions— whether treatment or preventative in nature—to each individual patient. Taking advantage of high performance computer capabilities, artificial intelligence (AI) algorithms can now achieve reasonable success in predicting risk in certain cancers and cardiovascular disease from available multidimensional clinical and biological data. In contrast, less progress has been made with the neurodevelopmental disorders, which include intellectual disability (ID), autism spectrum disorder (ASD), epilepsy and broader neurodevelopmental disorders. Much hope is pinned on the opportunity to quantify risk from patterns of genomic variation, including the functional characterization of genes and variants, but this ambition is confounded by phenotypic and etiologic heterogeneity, along with the rare and variable penetrant nature of the underlying risk variants identified so far. Structural and functional brain imaging and neuropsychological and neurophysiological markers may provide further dimensionality, but often require more development to achieve sensitivity for diagnosis. Herein, therefore, lies a precision medicine conundrum: can artificial intelligence offer a breakthrough in predicting risks and prognosis for neurodevelopmental disorders? In this review we will examine these complexities, and consider some of the strategies whereby artificial intelligence may overcome them.

## Introduction

A principle tenet of precision medicine is that subpopulations may be reasonably identified who differ in their disease risk, prognosis and response to treatment due to differences in underlying biology and other characteristics. The availability of multidimensional datasets that capture such variation can be ‘trained’ using artificial learning algorithms to find the cryptic phenotypic or genotypic structures, discussed subsequently, to then predict risk of disease, treatment response, prognosis and other outcomes in individual patients based on their own characteristics. The realization of this will offer clinicians the opportunity to more carefully tailor interventions—whether disease modifying or preventative in nature—to individual patients, contrasting with the current inductive process of symptom classification, and sometimes vague and inexact process of treatment decisions. One challenge of precision medicine is the high-performance computing requirements to process multidimensional datasets. However, computer capabilities have grown exponentially in recent years, and the integrated efforts of the international scientific community have made available large multidimensional biological and clinical datasets.^1–5^ Recently, prediction algorithms utilizing artificial intelligence approaches for cancer^6–9^ and cardiovascular disease^10,11^ have shown promising results, predicting disease risk with a higher degree of precision.

In part, of course, success is predicated on the availability of accurate biological measurements, adequate quantification of relevant environmental factors and, from a genomic perspective, the identification of variants of known penetrance. Realizing a similar approach to the group of disorders of brain development termed ‘neurodevelopmental disorders’ (NDD) has a number of obstacles.^12^ The NDDs are a group of early childhood onset disorders that impact different domains of cognitive development, motor function and other higher brain functions, and are lifelong in nature. Among the NDDs are severe disorders that impact multiple domains of cognitive functioning, such as intellectual disability (ID), as well as severe and pervasive disorders of social communication (autism spectrum disorder, ASD), motor function and cognition (epilepsy encephalopathies), and behavioral regulation (attention deficit hyperactivity disorder, ADHD). Some NDDs, particularly single gene disorders with more severe cognitive and medical consequences, are very rare. ASD and ADHD in particular are now relatively common, and result in major functional impairment, in part related to the high rates of co-morbidity. Epilepsy is one such comorbidity, with 20% of people with ASD also receiving this diagnosis. Moreover, epilepsy itself is often neurodevelopmental, although can sometimes occur de novo in adulthood or later in life. NDD co-morbidities are common and can make diagnosis challenging. Moreover, there is a degree of overlap in phenotype between different disorders, and phenotypic variability between individuals with the same diagnosis.^13–15^ These complexities, often resulting in misdiagnosis or even missed diagnosis, are a major catalyst for the implementation of precision medicine. This is particularly so because, as a group, such disorders place a significant burden on healthcare. As such, early diagnosis and targeted therapeutic interventions to those who are most likely to benefit are universally agreed public health priorities.^16,17^

In this review, the ambition of precision medicine will be described, and success and implementation in medical practice so far will be briefly presented, with certain cancers and cardiovascular disease as examples of success. The neurodevelopmental disorders will then be introduced and their inherent etiological and clinical complexities. Importantly, whilst large, principally genomic and clinical, datasets are available pertaining to individuals with NDD, using these data to facilitate improved diagnosis, therapeutic intervention and clinical outcomes is not straightforward. We will discuss the issues of clinical heterogeneity, lack of diagnostic clarity and biological overlap that characterize the NDDs. We will consider in detail potential approaches to address this complexity using epilepsy as exemplification, and then describe the outlook for artificial intelligence as applied to NDDs.

### Precision medicine and artificial intelligence

**Fig. 1:**
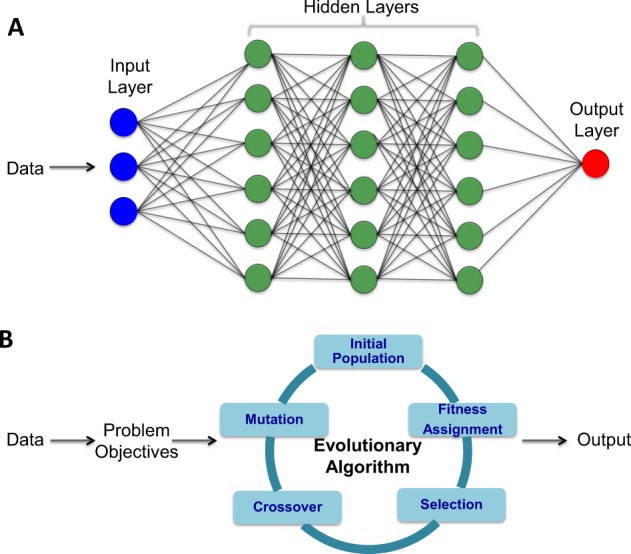
Most promising artificial intelligence algorithms. **a** Simplified illustration of a basic model of neural network that is widely used in deep learning algorithms and (**b**) the components of evolutionary algorithm framework for multi objectives optimization related problem.

Precision medicine is a healthcare pathway that employs numerous technologies to guide individually tailored diagnosis and treatment for patients. The availability of technologies, including high performance computing (HPC), as well as large biological datasets, are critical for the implementation of a precision medicine pathway that has the power to impact on healthcare. At the center of this strategy is a set of computer algorithms that identify patterns in multidimensional datasets that are then used to predict or optimize based on the availability of similar data on individual patients. Artificial intelligence algorithms apply learning strategies based on classification or pattern recognition to (multi-dimensional) input data in order to be able to predict from future datasets. In clinical medicine, for example, this may involve using results of pathological specimens to predict diagnosis and staging for the pathological specimen received on a new patient. There are many AI algorithms available, broadly defined according to whether they are supervised or unsupervised. Methods include the support vector, random forest, neural network and an evolutionary algorithm (EA). A brief overview of these methods is provided in Box 1. In recent years, both neural network driven machine learning and evolutionary algorithm (Fig. 1) have shown promising predictive potential for problems that are not solvable in polynomial algorithms (known as NP-hard problems).^18–20^ These two models can be adapted by providing input data in supervised, unsupervised or semi-supervised models (see definition in Box 2).

In the last few decades, digitization of medical health record added a massive amount of data related to healthcare. Large digitization initiatives like EMERGE network, and ‘All of Us’ by NIH, USA^25^; Electronic Health Record (EHR) initiatives by Canadian Institutes of Health Research,^26^ National Health Service, UK^27^ are some of the world’s largest electronic health record databases. The application of AI algorithms will be greatly benefitted from these large digitization efforts that can help establish genotype-phenotype relationship for genetic diseases and have the potential to infer numerous phenotypic correlations and associations. Of course, collecting large scale digital data will only be helpful if the data comprise relevant clinical information to model AI algorithms.

The application of AI in medicine is a burgeoning area of development in light of the major impact it could potentially have on healthcare provision. The application of machine learning in medical imaging on skin lesions^6^ and treatable retinal diseases^1^ has been the most impactful, and demonstrates the potential for this technology in medical practice. Deep learning algorithm to diagnose heart attack using 549 ECG records shows a sensitivity of 93.3% and specificity of 89.7%, outperforming cardiologists.^28^

Recently, DNA sequencing technology adopted machine learning to read out long stretches of DNA fragments from digital electronic signaling data. Long read technologies are important to resolve repetitive regions in the genome and detect complex structural variants. The current short reas technology can not resolve these issues and it is still unknown the disease risk contribution from repetitive region and structural variation of the genome. Nanopore sequencing technology in particular uses a neural network based deep learning method to ‘call’ DNA bases from the electronic signal produced by the nanopore flow cells. This method has accuracy over 98% and can produce mega base long DNA reads.^29^ There has been an attempt to use AI in the clinical classification of genomic variation, based on the characterization of non-coding variants^30^ splicing code,^5^ DNA/RNA binding proteins^2^ and non-coding RNA (ncRNA)^31^ using large-scale molecular datasets. Classifying mutations according to their clinical relevance is very complex due to the largely unknown penetrance of individual variants, (i.e., the probability of diagnosis given a particular variant is identified, or mathematically, P(disease+|variant+)) Moreover, high penetrance variants are largely infrequent, with those of low penetrance much more common. Although most of the variants are non-coding in our genomes, determining pathogenicity of rare or common non-coding variants still requires major advancement in genomics. It will require multidimensional biological data and the use of artificial intelligence approaches to decipher the pathogenicity. Furthermore, many penetrant variants are also known to have more than one clinical manifestation, known as pleiotropy, and many diagnoses are characterized by variable presentation (phenotypic heterogeneity). Despite this, however, recent deep learning methods have had some degree of success in the correct interpretation of phenotype and genomic data for disease risk in numerous types of cancer,^6,7,9,32,33^ diabetic retinopathy^34,35^ and pharmacogenomics.^36–38^ For example, in discriminating lymph node metastases, 7 independent deep learning implementations showed greater discrimination power (i.e., in relation to pathological versus non-pathological) compared to 11 pathologists.^7^ The best deep learning algorithm performed with an area under the curve (AUC) of 0.99, compared to 0.88 for ‘best’ clinician-derived score. The specificity found to be similar between AI and the diabetic retinopathy expert, AUC 0.96 and 0.98, respectively.

### Neurodevelopmental disorders (NDDs)

Neurodevelopmental disorders have their onset early in childhood and impact on a variety of functional domains, including cognition and executive function, language and social function, and motor function and behavior control.^39–41^ A number of different diagnoses are subsumed within this category, including intellectual disability (ID),^42^ autism spectrum disorder (ASD),^4,43^ attention deficit hyperactivity disorder (ADHD),^44^ tic disorders, and other movement disorders.^45,46^ Epilepsy and other early onset brain disorders, with or without associated ID, are also classified as NDDs^47,48^ (Table 1). NDDs such as ASD and ADHD are common, lifelong disorders that affect males more commonly than females. In contrast, some syndromal NDDs, particularly single gene disorders, are individually very rare. As such disorders are often defined according to known biological abnormality (e.g., Down Syndrome, Fragile X syndrome, Tuberous Sclerosis) much of what we discuss in this paper is specific to common NDDs that are not defined according to known biology. Patients present with varying degree of severity, and comorbidity for two or more NDD diagnoses is common. With the exception of certain epilepsy syndromes (e.g., West Syndrome), which can be diagnosed more definitively based on the results of electroencephalography (EEG), most NDDs, including epilepsy itself, are diagnosed according to the presence of a threshold number of symptoms identified by direct observation or informant history. This is particularly problematic, as the availability of reliable information will vary from individual to individual, and even expert opinion can vary from clinician to clinician, such that diagnostic endorsement is often not definitive. Moreover, due to the developmental nature of this category of disorders, the clinical picture can vary over time,^49^ with symptoms becoming more or less severe as the child grows. The availability of a more stable and objective way to classify individuals with NDDs is clearly needed, but currently this fuzziness within the diagnostic pathway is a significant barrier for the implementation of precision medicine in neurodevelopmental disorders.

**Table 1:** Major neurodevelopmental disorders, prevalence, genetic inheritance, sex ratio, and genetic diagnostic yield.

Major neurodevelopmental disorders	Prevalence (approximately)	Sex ratio (male/female)	Genetic diagnostic yield (SNV, Indel and CNV)
Autism spectrum disorders	1.69%^CDC^	4:1	>40%^43,52^
Epilepsy	1.2%^119^	1:1	>45%^120,121^
Intellectual disabilities	1.7%^122^	2:1	>50%^123–125^
Single gene disorders	<1%	1:1, except for X linked mental retardation syndromes	100% (complete diagnosis)

*CDC* Centers for Disease Control and Prevention, USA

All the NDDs considered in this current discussion are principally genetic in etiology.^50^ For example, the early twin and family studies in ASD all supported a strong, heritable genetic component, and ASD and a lesser phenotype termed the Broader Autism Phenotype (BAP) do tend to run in families.^51^ Some individual cases may result from rare, highly penetrant mutations,^4,43,52^ some of which segregate in a Mendelian fashion. Some rare genetic syndromes, such as Fragile X^53^ and Tuberous Sclerosis,^54^ are associated with a number of NDDs. In contrast, however, most appear to result from a more complex genetic architecture that involves one or more genetic variants of variable penetrance interacting with other epigenetic mechanisms and environmental factors.^13,55,56^ Understanding this genetic complexity is important, and it is anticipated that technological developments, both in silico but also laboratory based, will help unravel this. What is also striking is their degree of overlap in common-SNP based genetic etiology,^57^ and pattern of differentially expressed genes. To date, over 250 genes have been reported to have strong association with NDD.^58^ A very small number of genes (*SCN2A*, *CHD8*, *STXBP1*) and loci (16p11.2 microdeletion, 15q13.3 microdeletion etc.) that are found to be enriched within NDD are still below the level of 1% frequency threshold.^48,59–61^ The current clinical genetic diagnostic yield for severe, syndromic NDDs associated with ID is approximately 40% and it is higher if genome sequencing data are available for other members (parents, siblings) of the family.^62^

In imaging studies, similarities in brain function evident from fMRI and diffusion tensor imaging also point to overlap at the level of intermediate phenotype between a number of NDDs such as ASD and ADHD.^63,64^ Studies have examined diagnosed individuals while performing different neuropsychological tasks in the scanner, and the regions and structures in the brain that are active have been elucidated. This overlap in intermediate phenotype also extends to other mental disorders that are of later onset but are also increasingly being seen from a developmental perspective, such as schizophrenia and bipolar affective disorder.^65^ However, at a clinical level, the phenotypes differ quite markedly. As we discuss subsequently, machine learning offers the opportunity to examine biological datasets in both a supervised and unsupervised manner, thereby providing both predictive models for diagnosis and treatment, as well as, theoretically, examining how multidimensional datasets may inform new models of classification. Specifically regarding classification, AI may offer new insight into how overlap at the biological level maps into disorders that are different clinically.

### Artificial intelligence in NDDs

**Fig. 2:**
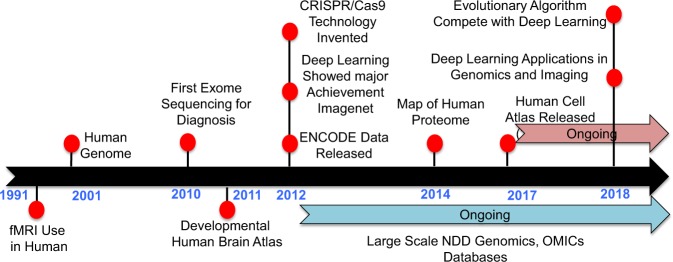
Historical milestones related to precision medicine and artificial intelligence.

The availability of fMRI that enabled the high-resolution capture of brain activity was a major milestone^66^ in NDD diagnosis and therapeutics in the 90s (Fig. 2). Since then, the human genome has been mapped^67,68^ and exome and whole-genome sequencing technologies have led to the detection of hundreds of disease causal genes and loci for ASD and other NDDs.^43,69–71^ Indeed, conducting exome or genome sequencing for newborn babies at high risk of genetic abnormalities is now becoming more frequent and cost effective.^72^ Subsequently, the advent of transcriptome sequencing dependent technologies led to the establishment of the Allen developmental human brain atlas^73^ in 2011, ENCODE database profiling the non-coding elements in the human genome^74^ in 2012, and the Human Cell Atlas^75^ in 2017. Multiple sequencing consortiums focussed on the NDDs were also started during the period of 2012 and 2014 with the aim of identifying disease-implicated variants, and making exome and WGS data available to the scientific community for further study.^52,70,76,77^ Bearing in mind that most identified genetic variation is of unknown pathogenicity, and little is known about functional consequences, the discovery of CRISPR/Cas as a gene editing tool in 2012 has allowed scientists to better characterize identified genetic variants.^78,79^

**Fig. 3:**
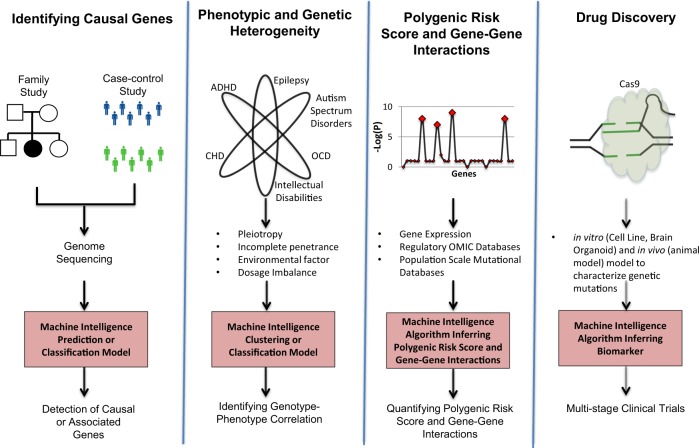
Complex unresolved problems in neurodevelopmental disorders that artificial intelligence algorithms can create an impact.

In recent years, artificial intelligence approaches has been used in autism spectrum disorder,^5,12,14,15^ epileptic encephalopathy,^80–82^ intellectual disability,^83–85^ attention deficit hyperactivity disorder (ADHD),^86^ and rare genetic disorders.^2^ In our discussion of AI in NDDs, three layers of analyses will be considered. The over-riding theme will be the application of these methods to multidimensional NDD biological datasets, and the complexities therein (Fig. 3).

AI approaches are critical for identifying causal genes and loci. Although the current genetic diagnostic yield (including copy number variation (CNV), single nucleotide variants (SNV), and indel) for severe, syndromal ID is around 50% (Table 1) we still do not know genes or loci for NDDs more generally, which includes many of the cases whether there is no ID and/or evidence of craino-facial dysmorphology.^58,62^ In addition, many identified loci are confounded by unknown penetrance, and, beyond bioinformatic prediction, do not have a strong evidential basis of support. De novo CNVs and SNVs and loss of function (LOF) mutations are certainly significantly enriched in individuals with NDD compared to typically developing controls.^4,71,76^ Unfortunately, bioinformatic prediction is still unable to confidently classify the more common missense mutations according to pathogenicity. Indeed, identifying causal genes from these ‘variants of uncertain significance’ (VUS) remains a major unresolved problem that does lend itself to a AI solution. Recently, the identification of *OTUD7A* as a pathological gene in the 15q13.3 microdeletion syndrome locus illustrated the power of integrating computational and molecular approaches to resolving causality in CNVs.^87^ Whilst this approach may certainly provide one solution, it is costly and, importantly, time intensive. Recently, post-zygotic mutations from blood and brain have been shown to be associated with ASD, epilepsy and ID.^48,60,88^ The abundance of neuron specific mutations has also been reported in the literature.^89^ What is unclear is the proportion of cells with potentially pathogenic postzygotic mutation. To comprehensively resolve genetic risk in relation to NDDs, there are other genomic regions that still need careful evaluation, such as non-coding variants, common variants and repeated sequences (over 40% of the entire genome). Moreover, an unsupervised learning approach, discussed below, may offer the opportunity to identify new patterns to data independent of these diagnostic categories.

Despite these limitations, AI approaches have recently shown reasonable success for improving genetic diagnostics in NDDs. As indicated above, one of challenging task is the correct classification of missense variants and Human Splicing Code,^5^ and DeepSEA^30^ showed very promising results in missense variant interpretations. The application of Human Splicing Code is one of the first machine-learning algorithms that shows convincing evidence of accurately classifying disease-causing variants, including those that are intronic. This method applies a Bayesian machine learning algorithm to model splicing dysregulation from a set of three or triplet exons. The method demonstrated pathogenic missense variants in ASD and in spinal muscular atrophy,^5^ including variants that had not previously been classifiable in this way. In contrast, DeepSEA is a deep learning based algorithm that predicts the noncoding variant effects de novo from sequence data. The model uses large-scale chromatin-profiling data, including transcription factor binding, DNase I sensitivity and histone-mark profiles to predict the functional consequences of a non-coding variant. These and other algorithms are performed independent of established diagnostic categories, and serve to enrich the information for each genomic element for incorporation into downstream analyses discussed subsequently. Such holistic approaches, therefore, resolve variant pathogenicity through the interpretation of multidimensional omics data in the context of different NDD diagnoses. The recent advent of long range sequencing technologies (e.g., Pacific Biosciences, Oxford Nanopore Technology and others) are producing high quality DNA sequencing data that allow repeated sequences to be resolved.

AI approaches are critical to elucidate hidden structure in phenotype and genetic heterogeneity. As indicated above, both phenotypic and genetic heterogeneity characterize NDDs. For example, 15q13.3 microdeletion syndrome impacts multiple domains of cognitive function and is associated with heterogenous phenotypes, including epilepsy/seizure (57%), speech delay (16%), and ASD (11%).^90^ There are hundreds of such CNVs with no straightforward mapping between manifested phenotypes and the variants/genes.^40,58^ Despite the possibilities, there remains the problem of phenotype, and in particular, the oversimplification of dichotomizing phenotypes such as ASD and ADHD into ‘caseness’. Variant information classified according to algorithms such as those defined above, as well as incorporation of other layers of biological data (neuroimaging, neurophysiology, neuropsychology), can be used to identify hidden structure in data, particularly if a unsupervised approach is used. These hidden structures may or may not map onto existing diagnostic categories, but, crucially, may be more closely aligned with endophenotypes, treatment response, prognosis and other clinical and outcome parameters. This discovery-driven approach may validate existing clinical diagnostic models of disorder classification, as well as potentially identify new models of classification that are driven principally (or, indeed, entirely) by the clustering of biological data. In NDDs in particular, diagnostic criteria have evolved significantly over time, principally due to a lack of clear, objective *sine qua non* for each disorder. Unfortunately, this evolution has seen the boundaries between disorders dissolving, milder forms being pathologized and discrete diagnostic categories morphing into spectra. Whilst biology does to some extent inform this nosological evolution, greater emphasis needs to be placed on using AI approaches on large-scale datasets to validate or challenge existing classification paradigms. Moreover, even if syndromes such as ADHD and ASD do truly exist as spectra, AI may be useful in identifying boundaries, perhaps informed by outcome and prognosis.

Although attempts with neural network deep learning approach showed that by combining fMRI with phenotypic data ASD classification can be improved,^91^ this is still predicated on the fundamental existence of a categorical diagnostic label, *viz*. ASD, that may not correctly capture the structure in the underlying data.^92^ Similarly, in epilepsy, EEG endophenotypes have been proposed^93^ and purely EEG-based classification of seizures have been investigated theoretically and clinically.^94^ However, none of these methods have been applied in a quantitative context, perhaps as the diagnosis of subtypes of epilepsy often rests heavily on qualitative EEG observations.

Methods are also needed that allow individuals to be assigned to more than one category in a probabilistic manner. For example, an individual may fall into diagnostic or endophenotypic category A with a probability of 0.9, and category B with a probability of 0.6 (we will highlight some examples of this in psychiatric conditions, and suggest that similar approaches can be taken in NDD). This closely reflects the reality of NDD symptom manifestation, whereby an individual with, say, ASD is also very likely to manifest ADHD or one of the other NDD diagnosis. In other words, co-morbid conditions may share an endophenotype that has clear diagnostic biomarkers. We need methods to both identify such diagnostic biomarkers, and to evaluate risk of different diagnostic categories for an given individual.

The availability of data-driven clinical diagnostic entities may also facilitate the triaging of patients in clinical practice. There is currently little opportunity to do this, as even well-designed screening instruments have limited reliability between sexes and across different ethnic groups. Although diagnostic criteria exist, there is much variation between clinicians on diagnostic thresholds used, which beyond the need for symptoms to impact on functioning are otherwise not explicitly written into these criteria. With the availability of data-driven categorization, there may be an opportunity, therefore, for the results of biological tests to inform who should be referred for further evaluation and or monitoring in a similar way to other medical tests. In addition, current diagnostic assessments for NDDs can be lengthy, and their multidisciplinary nature costly, leading to long waiting lists for children to receive diagnostic assessments. There is, therefore, a real opportunity for AI to automate some of the tasks in the diagnostic pathway and thereby have far reaching implications for clinical care and healthcare economics.

AI algorithms require major push to determine polygenic risk scores and gene-gene interactions. As we have stated before, hundreds of genes are involved in NDDs and the variability of gene variants (both common and rare) contributes to the overall pattern of brain function, as evidenced by fMRI and EEG, and phenotype at the clinical level. The risk prediction for each individual mutation is a complex process, as it will require a large number cases and controls to quantify significance. Similarly, predicting disease phenotypes from MRI and EEG data has similar problems, and incorporating data across these biological levels has challenges. For example, in epilepsy, siblings can share a very similar genetic and environmental background, but some develop epileptic seizures, while their siblings do not. In such cases, even the background EEG can appear very similar in siblings, but the exact factors causing one to have seizures is not well understood.

Even after quantifying risk factors, genetics lack well-established statistical or computational model that can utilize multiple variant or gene risk factors and combine them into a unified polygenic risk score. A neural network based approach on quantifying gene score for polygenic trait (i.e height) using single nucleotide polymorphism data showed promising improvements out performing previous methods.^95^

Polygenic risk prediction in NDDs remains problematic in light of the largely negative findings from underpowered genome-wide association studies (GWAS). Recently, a genome wide association study on large autism spectrum disorder and control cohort identified five common variants that confers very small risk factor.^57^ When the proportion from de novo risk factor is substantially large, it is still not clear to what extent common variants contributes into the genetic risk factor of neurodevelopmental disorders. Therefore, although common variants are likely to play an important role, particularly in relation to quantitative traits that merge with those in the population-at-large.

Regarding the inference of gene–gene interaction, the number of permutations and combinations involving all the genes in our genome is a massive exponential search space and there is no efficient algorithm that can infer gene-gene interactions involving more than few genes. Statistical significance for the large number of interactions also suffers from the impact of multiple testing thresholds.^96,97^ Although gene–gene interaction is likely a major contributor to the phenotypic variance of NDDs, there is currently no credible artificial intelligence algorithm able to cope with data on this scale. Certainly, a large number of genes can be simplified into a smaller number of protein–protein interactions or co-expression networks using traditional statistical model or algorithms, but as discussed subsequently, this is computationally NP-hard.^98^ Adding to this complexity, there may be significant overlaps between gene lists and/or protein/co-expression networks for different neurodevelopmental disorders, and so discriminatory classification of, say, ASD vis-à-vis schizophrenia adds further layers of complexity. This latter problem is perhaps the most important one to consider, as this will inform the translational capacity of the algorithm for precision medicine.

Although NDDs are mostly genetic in etiology, environment will still impact on genetically driven brain patterning, and therefore have the potential to influence disease severity. Recently multiple independent reports have showen an association between postzygotic mosaic mutations and autism spectrum disorders, intellectual disability, epilepsy and other NDDs.^48,60,88^ Currently the complex interactions between post zygotic mosaic mutations and environment is poorly understood.

AI approaches are at the frontier for therapeutic intervention and drug design. Currently, there are 51 food and drug administration (FDA) approved targeted gene specific drugs for neurology and psychiatric conditions. The advent of sequencing technology has principally been focussed on facilitating the implementation of early precision diagnostics. Precision therapeutics remains a major challenge for NDDs. Recently the advent of genome editing technologies (i.e., CRISPR/cas9), and antisense oligonucleotide therapy has allowed scientists to mimic cellular phenotype, and help identify precise molecular targets. For example, the application of CRISPR/cas9 helped knocked out a functional copy of *CHD8* gene in induced pluripotent stem cells (iPSCs). The knockout iPSCs showed differential expression of several thousands of genes in neural progenitors and impacts early differentiating neurons.^99^ CRISPR/cas9 or other cas family proteins are still error prone, and the experimental success is not highly accurate. The future hope is that CRISPR/cas9, antisense oligonucleotide therapy and gene therapy based technologies will allow us to detect precise target molecules for most of the mutated genes in NDD. This will eventually lead to the experimental pathway to design target molecules (i.e., antisense oligonucleotide, or siRNA) to inhibit or disrupt the faulty pathway. Such drug design will require a major push on artificial intelligence algorithm implementation.

Recently the idea of repurposing drug is becoming a major area of research as well. Finding out common pathway for approved drugs can benefit multiple diseases. Finding out these shared pathway relationship is complex and do not have enough molecular and genomic data to establish a connection. For example, mTOR pathway impacts a certain group of epilepsy individuals and the same pathway found to be dysregulated in tuberous sclerosis individuals.^54,100^ Hence, mTOR inhibitors have a great potential to impact treatment outcome for individuals with epilepsy carrying mTOR mutation or tuberous sclerosis related epilepsy.

### Challenges for artificial intelligence in relation to NDDs

There exist major complexities involving deep phenotypic and large scale omics data. Artificial intelligence will eventually radically transform healthcare delivery for patients with NDDs, but there are major hurdles that need to be resolved. For example, the modifying effect of environment is not well understood, but may explain disease discordance in monozygotic twins and the observation of different genetic risk factors in siblings. Identical variants may have different phenotypic consequences, and even recurrent large deletions, or bioinformatically predicted damaging mutations, may result in phenotype among some but no apparent consequences among others. Except in a few specific situations (for example, fetal alcohol syndrome,^101^ and microcephaly through infectious agents^102^), major environmental influences on NDDs and their contributions to phenotypic severity or heterogeneity are still unknown. Environmental impact is also highly likely to be a source for inducing post-zygotic mutations, recently shown to be associated with NDD.^48,60^ Moreover, these environmental factors may differ between countries and continents. As such, artificial intelligence might capture structure in data for one geographic location that is not relevant for disease risk in another location. Unsupervised AI models (Box 2) can be utilized to identify previously unknown sub-structures within NDD cases based on environmental factors that are local population (Fig. 3).

On the technical side, similar problems also arise due to different methods, tools, and protocols being used to collect data. For example, reliability and reproducibility of neuroimaging findings depend hugely on many experimental factors.^103^ Similarly, population scale omics data suffer from batch effect and technology specific biases.^104,105^ Thus, although large databases may be available for machine learning approaches, great care has to be taken in the quality and comparability of datasets used. Otherwise, any structure and information extracted from the data using artificial intelligence may be completely trivially driven by the composition of the data.

Omics data are necessarily multidimensional, and characterized by a large computational burden.^106–108^ Compounding this, with the advent of single cell genomics, the genomic architecture of NDDs is becoming apparent, and the formidable challenge this introduces to the understanding of disease pathophysiology. There are a large number of somatic variants that have been identified that have the potential to impact phenotypic severity. For example, there is evidence of certain somatic mutations associated with autism spectrum disorder, microcephaly, and epilepsy.^48,60,88^ Recent analysis has also shown that up to 40% of neurons could have a large mega base scale copy number variation.^109^ Single cell genomics has also identified private somatic mutations within each neuron.^89^ Although the contribution of somatic mutation to disease risk is not well understood, this particular type of mutation will add unpredictable variance within machine learning approaches and will impact replication significantly.

Lack of proper training datasets (control and case) and complexity on interpretability are two major issues in AI. Although collaborative initiatives have resulted in the growing availability of datasets that can be used for training, there is still a need for larger, more complete biomedical datasets that are representative of different populations and tissues. Initiatives such as 100,000 Genomes Consortium, MSSNG,^76^ The Simons Foundation Autism Research Initiative,^110^ The Exome Aggregation Consortium,^111^ ENCODE Project Consortium,^74^ The Genotype-Tissue Expression Consortium,^112^ Allen Brain Atlas^73^ offer huge potential for training and testing AI algorithms, but only if the data are complete, and similar, robust, measures have been used across samples and tissues (Fig. 3). This is often an issue in NDDs where on the one hand it may be difficult to engage the participant in all potential assessments employed, and on the other hand different studies may have used different measures.

Clinical implementation of artificial intelligence algorithms should be informed by the needs of the healthcare practitioner and their patients. Most artificial intelligence algorithms work as a ‘black box’, which may raise concern among health professionals, and raise questions from clinical service users. To overcome this, gold standard AI protocols need to be established that can be understood by healthcare professionals. There is also a need to be transparent about the limitations of AI methods. For example, in genetic algorithms, it can be extremely difficult for clinicians to decipher how through random operations (i.e., mutation, crossover) and variables the model reaches fitness convergence for optimum solutions in a multidimensional search space.^113^ Ultimately, however, despite the complexity of different algorithms, statistical models and tests are used to favor or refute evidence (i.e., *p*-values, false discovery rates, area under the curve), which can be understood by many professionals working in a clinical setting.^114^

The datasets themselves, multidimensional in nature, will also have been collected from multidisciplinary experts who may not necessarily ‘talk the same language’. What one person may call ‘case’, therefore, may be ‘non-case’ to another, and genetic variants may similarly vary in their interpretation in relation to significance. In addition, pre-processing of data and, indeed, even the design of the original study itself from which data are being collated, may present additional confounds to data interpretation. Fortunately, scientific methods have become much more transparent in recent years, and accessing detailed information pertaining to the methods used is often readily available for datasets. Of course, it is equally important for such information to be transparent in relation to the use of AI methods themselves.

One of the major setbacks in NDDs is the paucity of available treatments. There has been a downshift in industry-sponsored trials of potential compounds for the treatment of different NDDs. This downshift includes issues related to drug design, the lack of positive control and replication. This is unfortunate, as we are now beginning to uncover different aspects of brain structure and function at the molecular level that are associated with phenotypic consequence. These compounds may be the focus for potential drug development, and their known pathophysiology may inform repurposing of existing compounds (Fig. 3). One of the central complicating factor in compound screening, is the three dimensional structure of proteins. The complexity of predicting the tertiary structure from polypeptide sequence is a computationally intractable problem.^98^ AI based prediction algorithms can overcome such barriers through rigorous training datasets of polypeptide sequences.

Genome editing, antisense oligonucleotide therapy are two major technologies that show promise in facilitating an understanding of biology and consequently addressing the paucity of available treatments for NDDs. Recently, the clustered regularly interspaced short palindromic repeats (CRISPR) system showed the ability to correct mutations in vitro^79^ and in vivo^115^ in numerous diseases. Unfortunately, the CRISPR-Cas9 system currently lacks target precision. Moreover, the blood–brain barrier is a major challenge to deliver CRISPR like editing system in vivo into the brain cells. Regarding the delivery of genome editing machineries, recent efforts on vector and non-vector based CRISPR system delivery shows limited success on breaking the blood-brain barrier.^116,117^ For NDD, future treatment options should implement AI based algorithms that can design genome editing or antisense oligonucleotide design tools that are compatible with the in vivo delivery mechanism. Without the integration AI based algorithms, the potential of precision medicine will not be fully realized in NDDs.

The paradigm shift promised by precision medicine will of course impact frontline healthcare staff, who will need training in its strengths and limitations, as well as the interpretation and translation of AI-driven knowledge into information that is clinically meaningful for patients. The healthcare sector will need to build its high performance computation (HPC) capacity, and innovators will need to devise sophisticated AI-platforms. Consideration will need to be given to protection of data and the legal framework by which such data are stored and shared. Indeed, such considerations need to be happening significantly before any implementation, meaning that even now such discussions should be taking place. The sensitive nature of healthcare (and social care) information demands an absolute watertight system both in terms of storage and sharing, but also algorithm performance. Having one’s data breached, or being given someone else’s clinical information should not happen. Moreover, being given an incorrect diagnosis as a result of algorithmic failure should also never happen, particularly as it may be more difficult to identify than clinician error.

### Opportunities for ML in NDDs

The challenges described above, both in terms of the vicissitudinous nature of diagnostic labels that are poorly defined in the first place, and the high levels of variability observed across multiple levels of biological measurements, may also be reframed as real opportunities for ML. At its most radical, a completely unsupervised approach may identify new, and more biologically meaningful, diagnostic categories. One paradigm would see clinical and biological data pooled together, or alternatively biological data could ‘drive’ the generation of new diagnostic entities. This is very different from existing uses of ML which are predicated on the existence of diagnostic categories such as ASD, ADHD, and others. This does, however, introduce the risk of ‘overfitting’ data. In addition, of course, throwing out the baby with the bathwater may not be entirely appropriate, and so we also advocate ML endeavors that attempt to use existing diagnostic constructs according to available underlying biological data. Some such approaches have been reasonably successful in correct diagnostic assignment, and are more immediately implementable in clinical practice, as current treatment algorithms are very much focussed on these very diagnostic categories. Natural language processing (NLP) is another emerging field of machine intelligence that can automatically transform clinical text into structured clinical data.^118^ NLP algorithms can analyze digital health records and psychiatric notes to identify relatedness among patients’ phenotypes and their associated genetic markers. Although scientists have been working on NLP algorithms for the last few decades, significant improvements are still required from extracting text to understanding the clinical and biological relevance. ML approaches may be used to parse clinical text in the form of diagnostic reports. It is fairly standard practice for NDD diagnoses to be made following detailed assessment comprising clinical interview and objective testing such as IQ and other aspects of cognition. These assessments are typically summarized as a detailed report. Such reports contain rich data, that, theoretically, could be extracted using a suitable algorithm. This approach is based on the valid assumption that such data contain the unique insight of expert clinicians that might otherwise be overlooked in more formal measurements, or using pure biological data.

Analyzing data across multiple levels of biological function is also an attractive proposal in ML. By way of example, historically EEG has shown variable success in the identification of distinct neurophysiological patterns of impairment between ASD and controls. In brief, there is no consistent pattern of brain activation in response to particular stimuli that consistently differentiates the ASD from the non-ASD brain. However, there is strong reason to continue to pursue EEG-identified biomarkers for brain disorders, because as a method it represents a cost-effective, objective way of facilitating diagnosis that could easily be implemented in the clinical setting. ML algorithms with the capacity to handle longer EEG tracings (24 h for example) may be one potential avenue for exploration. Alternatively, consideration might be given to the ways in which other biological signals, from fMRI perhaps, improves the interpretability of EEG signals.

Finally, NDDs are not static disorders, but evolve over time, and one of its biggest challenges is the unpredictable nature of the progression. Thus, there is considerable within-subject variability in how the disorder changes over time, which is often neglected in the context of research studies. The challenge is to understand the manifestation of wide ranges of phenotypes during developmental stages in an individual that arises from the same genetic and neuronal substrate.^39,47,58^ Application of artificial intelligence algorithm on longitudinal studies can be designed to capture the pattern of disease progression over time and the variability at the personal or sub-population level.

Bearing in mind how long it takes a compound to go from original identification to eventual therapeutic use, ML algorithms will also have a significant role to play in parsing the large volumes of data generated during drug-development, as well as prioritizing molecules based on their known biological properties. Integrating genomics within the artificial intelligence drug development algorithm will enhance the implementation of precision medicine for NDD. Genomic profile can add the sensitivity that the artificial intelligence algorithm requires to design drug at the individual or a sub population level. Treatment response in NDDs is one other area of healthcare delivery that could benefit from ML, and this extends to the management of mental health disorders more generally. Despite much research, predicting treatment responsiveness remains very poorly understood. This is particularly important as many treatments require a period of time before efficacy (or lack thereof) is established. Consequently, patients may remain essentially untreated for many weeks, months if identifying a successful drug requires several attempts. There will, of course, be many reasons why there is such large variation in treatment response, and ML offers the opportunity to identify structure in multidimensional data that captures such things as metabolism, absorption, and disorder characteristics (severity, comorbidity and so forth).

Box 1 Neural network is a model comprised of multiple layers of artificial neuron-based structures that are equipped with multilayer logistic regressions.^21^ The model consists of an input and output layer and the artificial neurons in between are known as hidden layers (Fig. 1a). Neural network is widely used for machine learning (ML) related problems (i.e., pattern recognition or classification). Evolutionary algorithm (EA) is another model that was also adapted from nature.^22^ EA is an effective optimization model that usually starts from the random assignment of an initial possible solution (known as a population) and progressively applies artificial genetic operators (mutation crossover etc.) to produce a new set of possible solutions in the subsequent generation (Fig. 1b). EA is well known for its capability in optimizing multiple objectives.^23^ Although deep learning is becoming more popular, in a recent paper a type of EA algorithm was shown to outperform a deep learning algorithm in classical gaming theory.^24^

## Conclusions

Artificial intelligence is already impacting healthcare, and it is hoped that some of the successes achieved so far in cancer and cardiovascular disease will also be seen in the NDDs. This will necessarily involve the integrated use of existing and new supervised and unsupervised learning approaches, as well as an HPC infrastructure that can manage the multidimensional nature of the emerging omics data. There needs to be major investment in new treatments that will map onto different disease categories and subcategories, and researchers need to step away from existing diagnostic constructs to embrace a more intermediate biologically driven level of phenotype that may map more neatly onto treatment response and clinical outcomes. The healthcare sector, which is already financially stretched, has a formidable task ahead: there needs to be a development of infrastructure, expertize in knowledge translation among healthcare professionals, and engagement with the service users themselves in developing new clinical pathways. Short term investment in ML will certainly have long term gains, both in terms of financial savings resulting from precision medicine, but also the ultimate improvement in the health of the population (Box 2).

Box 2 **Supervised AI Algorithm:** In supervised learning algorithm, the training data helps the algorithm learning a function that maps an input to an output based on known or labeled input-output pairs.

**Unsupervised AI Algorithm:** Unsupervised learning is a type of machine learning that involves unlabeled training data where the input-output relationship is not known and the algorithm infers patterns (or possible solutions) within datasets.

**Semi-supervised AI Algorithm:** Semi unsupervised learning is a type of machine learning that involves a mix of known and unknown training data that helps the algorithm to infer input-output relationships.
